# Supplementary figures and images for: Bioconversion of mango peels into itaconic acid through submerged fermentation and statistical optimization of parameters through response surface methodology
Source: PeerJ. 2024 Oct 18;12:e18188. doi: 10.7717/peerj.18188 (PMC11493022; doi:10.7717/peerj.18188)

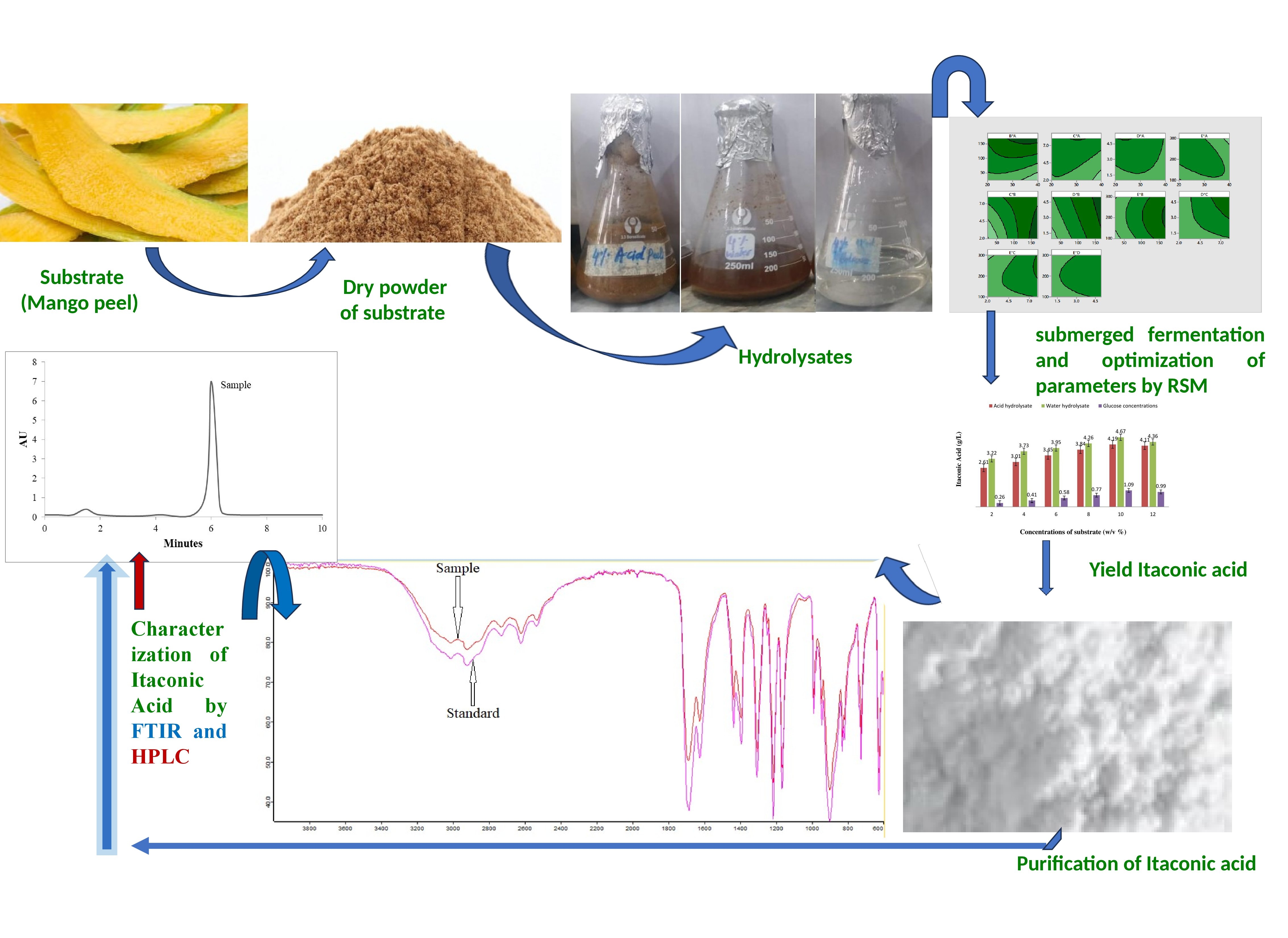

Supplement: Supplemental Information 2 [file peerj-12-18188-s002.jpeg]
